# Higher expression levels of SOCS 1,3,4,7 are associated with earlier tumour stage and better clinical outcome in human breast cancer

**DOI:** 10.1186/1471-2407-10-178

**Published:** 2010-04-30

**Authors:** Walid Sasi, Wen G Jiang, Anup Sharma, Kefah Mokbel

**Affiliations:** 1 St. George's University of London, London, UK; 2 Cardiff University, Cardiff, UK; 3 The London Breast Institute, The Princess Grace Hospital, London, UK

## Abstract

**Background:**

Suppressors of cytokine signaling (SOCS) are important negative feedback regulators of the JAK/STAT signaling pathway, and have been recently investigated for their role in the development of different cancers. In this study, we examined the expression of SOCS1-7 genes in normal and breast cancer tissue and correlated this with several clinico-pathological and prognostic factors.

**Methods:**

SOCS1-7 mRNA extraction and reverse transcription were performed on fresh frozen breast cancer tissue samples (n = 127) and normal background breast tissue (n = 31). Transcript levels of expression were determined using real-time PCR and analyzed against TNM stage, tumour grade and clinical outcome over a 10 year follow-up period.

**Results:**

SOCS1,4,5,6 and 7 expression decreased with increased TNM stage (TNM1 vs. TNM3 p = 0.039, TNM1 vs. TNM4 p = 0.016, TNM2 vs. TNM4 p = 0.025, TNM1 vs. TNM3 p = 0.012, and TNM1 vs. TNM3 p = 0.044 respectively). SOCS2 and 3 expression decreased with increased Nottingham Prognostic Index (NPI) (NPI1 vs. NPI3 p = 0.033, and NPI2 vs. NPI3 p = 0.041 respectively). SOCS7 expression decreased with higher tumour grade (Grade 3 vs. Grade 2 p = 0.037). After a median follow up period of 10 years, we found higher levels of SOCS1,2 and 7 expression among those patients who remained disease-free compared to those who developed local recurrence (p = 0.0073, p = 0.021, and p = 0.039 respectively). Similarly, we found higher levels of SOCS 2,4, and 7 expression in those who remained disease-free compared to those who developed distant recurrence (p = 0.022, p = 0.024, and p = 0.033 respectively). Patients who remained disease-free had higher levels of SOCS1 and 2 expression compared to those who died from breast cancer (p = 0.02 and p = 0.033 respectively). The disease free survival (DFS) and overall survival (OS) curves showed that higher levels of SOCS1, 3 and 7 were significant predictors of higher DFS (p = 0.015, p = 0.024 and 0.03 respectively) and OS (p = 0.005, p = 0.013 and p = 0.035 respectively). Higher levels of SOCS 4 were significant in predicting better OS (p = 0.007) but not DFS. Immunohistochemical staining of representative samples showed a correlation between SOCS1, 3, 7 protein staining and the SOCS1, 3, 7 mRNA expression.

**Conclusion:**

Higher mRNA expression levels of SOCS1, 3, 4 and 7 are significantly associated with earlier tumour stage and better clinical outcome in human breast cancer.

## Background

Signal transducers and activators of transcription (STATs) are intra-cytoplasmic proteins which are activated by phosphorylation to participate in gene control on a single tyrosine when cells encounter various extracellular cytokines, growth factors and hormones [1-3]. Seven STAT proteins have been identified to date; STAT1, 2, 3, 4, 5A, 5B, and 6 [1]. STAT binding to the Janus Kinase (JAK) receptor-associated tyrosine kinases occurs through the STAT SRC-homolgy-2 (SH2) domain resulting in their subsequent dimerization, phosphorylation and activation. Phospho-STATs (pSTATs) then move into the nucleus to be involved in the complex mechanism of signal transduction which leads to transcription of specific proteins. STAT3 plays a pleomorphic role in signal transduction. It typically acts as an oncogene. STAT3 regulates expression of VEGF and is associated with angiogenesis and tumor progression[4]. Activation of STATs has been reported in many cancers; head and neck, breast, prostate, pancreas and leukemia[5-10]. Furthermore, STAT3 expression is reported to be correlated with lymph node metastasis [11-13], and higher expression of STAT3 and pSTAT3 indicates a worse prognosis [10,14-16]. More recent data have shown a controversial role of STATs in breast cancer. Walker et al have demonstrated that STAT5 and STAT3 mediate opposing effects on several key target genes, with STAT5 exerting a dominant role. Using a model system of paired breast cancer cell lines, they found that co-activation of STAT5 and STAT3 leads to *decreased *proliferation and increased sensitivity to the chemotherapeutic drugs paclitaxel and vinorelbine compared with cells that have only STAT3 activation [17].

The activation of the JAK/STAT pathway is negatively regulated by a classical feedback loop through a group of proteins named Suppressors of cytokine signaling (SOCS) which are rapidly induced by activated STATs [18]. SOCS family consists of 8 proteins (SOCS1-7 and a cytokine-inducible SH2-containing protein or CIS), each has a central SH2 domain, an amino-terminal domain of variable length and sequence, and a carboxy-terminal 40-amino-acid molecule known as the SOCS box. SOCS molecules act to block the cytokine signal either by direct inhibition of JAKs (e.g. SOCS1), or by binding to the tyrosine-phosphorylated receptor to prevent binding of other SH2 and PTB domain-containing signaling proteins such as STATs (e.g. CIS), or by both mechanisms (e.g. SOCS3) [19].

SOCS proteins (e.g. CIS) are also involved in a fourth inhibitory mechanism which is their ability to accelerate proteasome-mediated destruction of the activated cytokine-receptor complex [19,20]. These inhibitory regulators not only attenuate the signal from the activated cytokine receptor itself, but can also decrease cell sensitivity to other cytokines and hormones, such as PGE2, insulin, and prolactin [21-23].

Furthermore, other reports showed a role for the SOCS proteins in regulating other signal transduction molecules (e.g. FAK, IRS, p65, GR) that are highly relevant to carcinogenesis [24-28]

Few studies have reported the role of SOCS proteins in breast carcinoma, and their possible influence on tumour growth and differentiation, but the general trend of their results indicates a favorable role of SOCS in attenuating cytokine signaling implicated in breast cancer growth. Knowing that SOCS gene promoter hypermethylation is an important mechanism in SOCS gene silencing and subsequent deregulation of the JAK/STAT signaling, Sutherland et al found promoter hypermethylation and silencing of SOCS1 (but not of SOCS2 or SOCS3) to occur in 9% of breast cancers and various degrees of SOCS transcription in several breast carcinoma cell lines, and suggested that silencing of specific SOCS genes in breast, may augment cytokine responsiveness, thereby contributing to oncogenesis [29]. Another report by Farabegoli et al demonstrated that SOCS2 expression was associated with high differentiation and a low proliferation rate in a group of 50 archival breast cancer samples, and was found to inversely correlate with several proliferation markers such as cyclin A and Ki-67 [30]. Haffner et al found an inverse correlation between SOCS2 mRNA expression and histopathological grade, and ER positive tumours exhibited higher SOCS 2 levels [31]. The later study has also reported SOCS2 as a significant independent predictor of good prognosis. A further study by Nakagawa et al has proved a lower SOCS1 and 3 expression in specimens with blood vessel invasion and in samples from patients with lymph node positive disease with the lowest SOCS3 expression found in patients with more than 4 lymph nodes involved in the metastatic process [32].

In view of this potential importance of SOCS expression in regulation of growth and development of breast cancer, and in view of the favorable data from previous reports, we have examined the expression of SOCS1-7 in a group of archival normal and breast cancer tissue specimens, and correlated their expression levels with several clinical, pathological and prognostic parameters within the same cohort of patients.

## Methods

### Patients and samples

Institutional guidelines, including informed consent, were followed. Ethical approval was obtained from St George's Healthcare NHS Trust ethics committee. Primary breast cancer tissues (n = 127) and matched non-neoplastic mammary tissue (from the same mastectomy specimens) (n = 31) were collected immediately after surgical excision and stored at -80°C for future use. Details of histology were provided by independent specialist pathologist using hematoxylin and eosin (H & E) - frozen sections. Where normal non-neoplastic tissues were used, no tumour cells were found in the sections. All tissues were randomly numbered and the details were only made known after all analyses were completed. All patients were treated according to local algorithms of management following a multidisciplinary discussion. Patients treated with breast-conserving surgery received adjuvant radiotherapy. Those with hormone- sensitive malignancy received tamoxifen. Fit patients with node-positive breast cancer or hormone-insensitive large and/or high grade cancer were offered adjuvant chemotherapy. Medical notes and histology reports were used to extract clinico-pathological data (Table 1).

**Table 1:** Clinical and pathological data (follow up period 120 +/- 6 months)

Parameter	Category	Number
**Node status**	Node positive	54
	Node negative	73
**Tumour grade**	1	24
	2	43
	3	58
**Tumour type**	Ductal	98
	Lobular	14
	Medullary	2
	Tubular	2
	Mucinous	4
	Others	7
**TNM staging**	1	70
	2	40
	3	7
	4	4
**Outcome**	Disease free	90
	Alive with metastasis	7
	With local recurrence	5
	Died of breast cancer	16
	Died of unrelated disease	9

Note: missing values reflect discarded/uninterpretable values.

### Materials

RNA extraction kits and reverse transcription kits were obtained from Sigma-Aldrich Ltd (Poole, Dorset, England, UK). The PCR primers were designed using Beacon Designer (Palo Alto, CA, USA) and synthesized by Sigma-Aldrich. Custom made hot-start Master mix for quantitative Polymerase Chain Reaction (PCR) was obtained from Abgene (Surrey, England, UK) [33,34].

### Tissue processing, RNA extraction and cDNA synthesis

Frozen sections of tissue were cut at a thickness of 5 - 10 μm and kept for routine histological analysis. Additional 15-20 sections were mixed and homogenized using a hand-held homogenizer in ice-cold RNA extraction solution. The concentration of RNA was determined using UV spectrophotometry. Reverse transcription was carried out using a reverse transcription kit with an anchored oligo (dT) primer supplied by Abgene, using 1 μg of total RNA in a 96-well plate. The quality of cDNA was verified using β-actin primers (Table 2).

**Table 2:** SOCS1 - 7 Primers

SOCS1	
Forward	GATGGTAGCACACAACCAG
Z Reverse	ACTGAACCTGACCGTACAGAGGAAGAGGAGGAAGGTT
SOCS2	
Forward	GGATGGTACTGGGGAAGTAT
Z Reverse	ACTGAACCTGACCGTACATGCGAGCTATCTCTAATCAA

SOCS3	
Forward	CCACTCTTCAGCATCTCTGT
Z Reverse	ACTGAACCTGACCGTACAATCGTACTGGTCCAGGAACT

SOCS4	
Forward	GGCAGTGTTTTCCAATAAAG
Z Reverse	ACTGAACCTGACCGTACAAGGTGGGAAAGGACACTTAT

SOCS5	
Forward	AGTCAAAGCCTCTCTTTTCC
Z Reverse	ACTGAACCTGACCGTACACATTTTTGGGCTAAATCTGA

SOCS6	
Forward	CCTTACAGAGGAGCTGAAAA
Z Reverse	ACTGAACCTGACCGTACACGAACAAGAAAAGAACCATC

SOCS7	
Forward	CAGGCCCTGAATTACCTC
Z Reverse	ACTGAACCTGACCGTACAGAGGTTGCTGCTGCTGCT

β-Actin	
Forward	ATGATATCGCCGCGCTCGTC
Reverse	CGCTCGGTGAGGATCTTCA

#### Immnunohistochemical staining for frozen sections

The SOCS3 protein was detected by a rabbit polyclonal antibody (H-103; Santa Cruz Biotechnology Inc, Santa Cruz, California, USA). For SOCS7 protein detection, goat polyclonal antibody was used (C-19; Santa Cruz Biotechnology Inc, Santa Cruz, California, USA). The procedure was similar to that previously reported, with minor modifications[34,35]. Briefly, the frozen sections of breast tumour and non-neoplastic breast tissue were cut at a thickness of 5 μm using a cryostat. The sections were mounted on microscope slides, air-dried and then fixed in a mixture of 50% acetone and 50% methanol for 20 min. The sections were then placed in a Menapath autowash buffer for 5-10 min to rehydrate. Sections were incubated for 20 min in a horse serum blocking solution and probed with the primary antibody (1:50 dilution Anti-SOCS3, and Anti-SOCS7) at room temperature for 1 h. Following extensive washings, sections were incubated for 30 min in the secondary horse serum. Following washings, Avidin biotin complex (Vector Laboratories Ltd.) was then applied to the sections, followed by extensive washings. Diaminobenzidine chromogen (Vector Laboratories Ltd.) was then added to the sections, which were incubated in the dark for 5 min. The sections were then dehydrated in ascending grades of methanol before clearing in xylene and mounting under a coverslip before initial photographs were taken. A rehydration process was then performed with descending grades of methanol before removing the coverslip and counterstaining in Mayer's hematoxylin for 1 min.

The process of dehydration and mounting was then repeated. Staining intensity was semiquantified using a method established in our laboratory [34], which was modified and based on that reported by Fidler and colleagues[35]. Briefly, gray-scale digitized images were imported into the Optimas software (Optimas 6.0, Optimus Corp., Bothell, WA, USA). Control staining (without primary antibody) was used for the extraction of the background staining.

### Quantitative analysis

The level of SOCS1 - 7 transcripts from the above prepared DNA were determined using real-time quantitative PCR based on the Amplifluor technology, modified from a method reported previously [32]. The PCR primers were designed using Beacon Designer software, but an additional sequence, known as the Z sequence (5'-ACTGAACCTGACCGTACA-3') which is complementary to the universal Z probe (Intergen Inc., Oxford, UK) was added to the reverse primer. The primers used for each SOCS are detailed in table 2. The reaction was carried out using Hotstart Q-master mix (Abgene), 10 pmol of the specific forward primer, 1 pmol of reverse primer which had the Z sequence, 10 pmol of FAM- tagged probe (Intergen Inc.), and cDNA from 50 ng of RNA. The reaction was carried out using the IcyclerIQ (Bio-Rad Ltd, Hemel Hempstead, England, UK), which is equipped with an optic unit that allows real-time detection of 96 reactions, under the following conditions: 94°C for 12 min and 50 cycles of 94°C for 15 sec, 55°C for 40 sec, and 72°C for 20 sec. The levels of the transcript were generated from a standard plasmid contained the specific DNA sequence that was simultaneously amplified with the samples. The levels of SOCS1-7 gene expression were then normalized against the housekeeping gene β-actin, which was already quantified in these specimens. Absolute quantification analysis was used and the resulting expression data were presented as ratios to β-actin [36]. The primers used for β-actin are detailed in table 2. With every PCR run, a negative control without a template and a positive control known cDNA reference sample, were included.

### Statistical analysis

The Mann-Whitney *U*-test (comparison of median copy number) and two-sample *t*-test (comparison of mean copy number) were used for statistical analysis of absolute and normalised gene copy number. The transcript levels within the breast cancer specimens were compared to normal background tissues and analyzed against conventional pathological parameters and clinical outcome over a 10 year follow-up period.

Within the tumour samples, the correlation between SOCS1 - 7 and downstream regulated genes was examined using Pearson's correlation coefficient. In each case the true copy number was used for statistical analysis and hence the samples were not classified as positive or negative. The statistical analysis was carried out using Minitab version 14.1 (Minitab Ltd. Coventry, England, U.K.) using a custom written macro (Stat 2005.mtw).

For purposes of the Kaplan-Meier survival analysis, the samples were divided arbitrarily into two groups, 'high transcript level' or 'low transcript level', for each SOCS gene. The cut-off was guided by the Nottingham Prognostic Index (NPI) value, with which the value of the moderate prognostic group was used as the dividing line at the start of the test. Disease Free Survival (DFS) and Overall Survival (OS) analyses were performed using SPSS version 12.0.1 (SPSS Inc. Chicago, IL, USA). For multivariate analysis using the Cox regression model, PASW Statistics 18 Software (Chicago, IL, USA) was used.

## Results

### SOCS1 - 7 mRNA expression by Quantitative PCR

The SOCS1-7 expression profiles were determined both in absolute terms and normalised against β-actin. All SOCS1-7 expression profiles showed no significant differences in association with different responses to chemotherapy in the patient cohort.

### SOCS1 (Table 3)

SOCS1 was found to be expressed in both normal/benign breast tissue and breast cancer specimens. No significant difference was found between SOCS1 expression in breast cancer specimens and its expression in normal background tissue.

**Table 3:** SOCS 1 - 3 Mean mRNA expression levels

Patient and tumour characteristics	SOCS1 Mean (SD)	P	SOCS2 Mean (SD)	P	SOCS3 Mean (SD)	P
ER Status						
ER(-) vs. ER(+)	47(165) vs. 18.4(38.4)	0.18	329585 (1361524) vs. 241896 (1097499)	0.72	0.6 (3.1) vs. 3.2 (10.7)	0.16

Tumor Grade						
Grade 1 vs. 2	90(189) vs. 41(163)	0.33	335817 (1426927) vs. 44859 (128040)	0.37	4 (13.5) vs. 0.8 (4.1)	0.32
Grade 1 vs. 3	90(189) vs. 10.3(53.6)	0.08	335817 (1426927) vs. 512760 (1654661)	0.65	4 (13.5) vs. 0.7 (3.1)	0.3
Grade 2 vs. 3	41(163) vs. 10.3(53.6)	0.26	44859 (128040) vs. 512760 (1654661)	0.04	0.8(4.1) vs. 0.7 (3.1)	0.9

NPI						
NPI 1 vs. 2	20.1(86.4) vs. 51(178)	0.32	495133 (1685902) vs. 66274 (241181)	0.06	1.2 (7.7) vs. 2.1 (6)	0.52
NPI 1 vs. 3	20.1(86.4) vs. 58(148)	0.35	495133 (1685902) vs. 12414 (25254)	0.03	1.2 (7.7) vs. 0.1 (0.2)	0.26
NPI 2 vs. 3	51(178) vs. 58(148)	0.88	66274 (241181) vs. 12414 (25254)	0.18	2.1 (6) vs. 0.1 (0.2)	0.04

TNM						
TNM 1 vs. 2	53(170) vs. 20.5(67.1)	0.19	157325 (679157) vs. 364680 (1454925)	0.42	1.9 (8.4) vs. 0.9 (3.6)	0.79
TNM 1 vs. 3	53(170) vs. 5.7(13)	0.039	157325 (679157) vs. 1180151 (3122352)	0.42	1.9 (8.4) vs. 0.3 (0.9)	0.17
TNM 1 vs. 4	53(170) vs. 3.2(5.3)	0.027	157325 (679157) vs. 3256 (6508)	0.08	1.9 (8.4) vs. 0.2 (0.2)	0.12
TNM 2 vs. 3	20.5(67) vs. 5.7(13)	0.23	364680 (1454925) vs. 1180151 (3122352)	0.52	0.9 (3.6) vs. 0.3 (0.9)	0.39
TNM 2 vs. 4	20.5(67) vs. 3.2(5.3)	0.13	364680 (1454925) vs. 3256 (6508)	0.14	0.9(3.6) vs. 0.2 (0.2)	0.23
TNM 3 vs. 4	5.7(13) vs. 3.2(5.3)	0.67	1180151(3122352) vs. 3256 (6508)	0.36	0.3(0.9) vs. 0.2 (0.2)	0.68

Survival						
DF vs. LR	48(154) vs. 1.2(3.2)	0.007	360706 (1370000) vs. 3499 (9061)	0.02	1.5 (7.3) vs. 0.8 (2.1)	0.55
DF vs. DR	-	-	360706 (1370000) vs. 3897(8714)	0.02	-	-
DF vs. D	48(154) vs. 7.4(13.3)	0.02	360706 (1370000) vs. 27239 (81017)	0.03	1.5 (7.3) vs. 0.4 (1.1)	0.22

SOCS 1 - 3 Mean mRNA expression levels in a cohort of 128 breast cancer patients; a comparison between subgroups with different tumour grade, NPI score, and TNM stage. SD: Standard deviation, DF: Disease free survival, LR: Local disease recurrence, DR: Distant disease recurrence, D: Death from breast cancer

The expression of SOCS1 mRNA did not significantly differ with increasing Nottingham Prognostic Index (NPI) or between normal background breast tissue and tumour tissues of patients with different NPI levels. The expression of SOCS1 mRNA was demonstrated to significantly decrease with increasing TNM stage; TNM-1 vs. TNM-3 [mean copy number 53 vs. 5.7, 95% CI (2, 91.5), p = 0.039], and TNM-1 vs. TNM-4 [mean copy number 53 vs. 3.2, 95% CI (6, 93.3), p = 0.027].

Transcript levels did not significantly differ with different tumour grades or with oestrogen receptor (ER) status.

After a median follow up of 10 years, we found SOCS1 mRNA expression levels to be higher among women who remained disease free compared to those who developed local recurrence [mean copy number 48 vs. 1.2, 95% CI (13, 81.2), p = 0.0073], and compared to those who died from breast cancer [mean copy number 48 vs. 7.4, 95% CI (6, 75.6), p = 0.021].

SOCS1 expression was also higher among patients who remained disease free compared to all other patients who developed any type of recurrence (local or distant) or died from breast cancer during the same follow up period [mean copy number 48 vs. 4.3, 95% CI (10, 78.2), p = 0.012].

**Figure 1:**
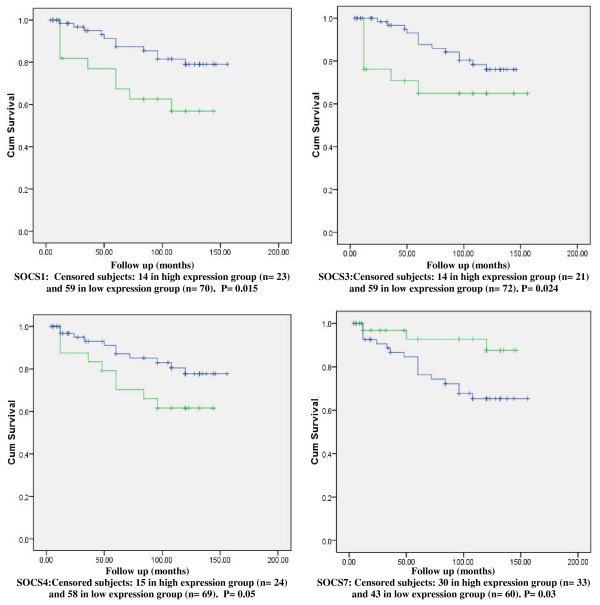
**Kaplan Meier Disease Free Survival (DSF) Curves for SOCS1,3,4,7**. Survival times are expressed as mean number of months with 95% confidence interval.

**Figure 2:**
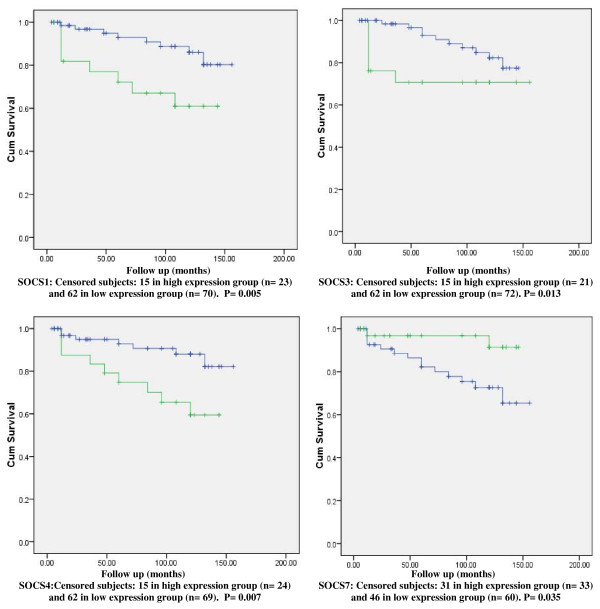
**Kaplan Meier Overall Survival (OS) Curves for SOCS1,3,4,7**. Survival times are expressed as mean number of months with 95% confidence interval.

There was a trend for tumours with lower SOCS1 expression levels to be associated with shorter DFS and OS times. The DFS and OS curves for women with tumours which were classified as having 'high levels' of SOCS1 transcripts were found to differ significantly from those of their 'low level' counterparts. The survival curves show higher levels of SOCS1 were of significant benefit in predicting higher DFS (p = 0.015) and better OS (p = 0.005). (Figures 1 and 2)

### SOCS2 (Table 3)

SOCS2 was found to be expressed in both normal/benign breast tissue and breast cancer specimens. No Significantly different levels were found in breast cancer specimens compared to normal background tissue. The expression of SOCS2 mRNA was found to decrease with increasing NPI; NPI-1 vs. NPI-3 [mean copy number 495133 vs. 12414, 95% CI (39141, 926297), p = 0.033], but was found to significantly *increase *with higher tumour grade; grade 2 vs. grade 3 [mean copy number 44859 vs. 512760, 95% CI (-921508, -14293), p = 0.043]. SOCS2 transcript levels were not significantly different with ER status.

After a median follow up of 10 years, we found SOCS2 mRNA expression levels to be higher among women who remained disease free compared to those who developed local recurrence [mean copy number 360706 vs. 3499, 95% CI (54131, 660284), p = 0.021], compared to those who developed distant recurrence [mean copy number 360706 vs. 3897, 95% CI (53710, 659909), p = 0.022], and compared to those who died from breast cancer [mean copy number 360706 vs. 27239, 95% CI (27587, 639348), p = 0.033]. SOCS2 expression was also higher among those who remained disease free compared to all other patients who developed any type of recurrence (local or distant) or died from breast cancer during the same follow up period [mean copy number 360706 vs. 16359, 95% CI (40447, 648249), p = 0.027].

The DFS curve for women with tumours with 'high levels' of SOCS2 transcript was not found to differ significantly from that of their 'low level' counterparts. Similarly, data from patients with tumours with higher transcript levels showed no statistically significant association with OS.

### SOCS3 (Table 3)

There was neither a significant difference in SOCS3 mRNA expression levels between cancer tissue and normal background tissue nor there was a significant difference in the transcript levels with different tumour grades or TNM classes. There was, however, a significantly lower SOCS3 transcript levels with higher NPI; NPI-2 vs. NPI-3 [mean copy number 2.14 vs. 0.092, 95% CI (0.09, 4.003), p = 0.041]. The DFS curve for women with tumours which were classified as having 'high levels' of SOCS3 transcript was found to differ significantly from that of their 'low level' counterparts, showing a significant benefit in predicting better DFS (p = 0.024). Similarly, the OS curve of patients with tumours classified as having higher transcript levels predicted a better OS (p = 0.013). (Figures 1 and 2)

### SOCS4 (Table 4)

The expression of SOCS4 mRNA was demonstrated to decrease with increasing TNM stage; TNM-1 vs. TNM-4 [mean copy number 384 vs. 1.51, 95% CI (73, 691.9), p = 0.016], and TNM-2 vs. TNM-4 [mean copy number 180 vs. 1.51, 95% CI (1, 355.5), p = 0.049] but did not significantly differ with tumour grade or with NPI.

**Table 4:** SOCS 4 - 7 Mean mRNA expression levels

Patient and tumour characteristics	SOCS4 Mean (SD)	P	SOCS5 Mean (SD)	P	SOCS6 Mean (SD)	P	SOCS7 Mean (SD)	P
ER Status								
ER(-) vs. ER(+)	198 (834) vs. 296 (1035)	0.63	1669 (2950) vs. 1066 (2215)	0.25	450 (1209) vs. 2018 (5905)	0.13	2310 (8080) vs. 715 (1341)	0.11

Tumor Grade								
Grade 1 vs. 2	142 (540) vs. 554 (1468)	0.13	834 (2468) vs. 2164 (3270)	0.09	2110 (7427) vs. 968 (2171)	0.51	5459 (14250) vs. 1396 (2583)	0.22
Grade 1 vs. 3	142 (540) vs. 113 (380)	0.82	834 (2468) vs. 2076 (5191)	0.17	2110 (7427) vs. 475 (1267)	0.34	5459 (14250)	0.13
Grade 2 vs. 3	554 (1468) vs. 113 (380)	0.07	2164 (3270) vs. 2076 (5191)	0.92	968 (2171) vs. 475 (1267)	0.21	vs. 427 (1339) 1396 (2583) vs. 427 (1339)	0.037

NPI								
NPI 1 vs. 2	276 (1004) vs. 168 (513)	0.49	1742(2846) vs. 1715 (3064)	0.97	1117 (4564) vs. 687 (1610)	0.51	2740 (8754) vs. 518 (1189)	0.06
NPI 1 vs. 3	276 (1004) vs. 419 (1441)	0.72	1742 (2846) vs. 3132 (9006)	0.56	117 (4564) vs. 439 (1138)	0.31	2740 (8754) vs. 538 (1125)	0.07
NPI 2 vs. 3	168 (513) vs. 419 (1441)	0.52	1715 (3064) vs. 3132 ( 9006)	0.56	687 (1610) vs. 439 (1138)	0.53	518 (1189) vs. 538 (1125)	0.95

TNM								
TNM 1 vs. 2	384 (1208) vs. 180 (532)	0.25	1856 (3095) vs. 2328 (6035)	0.66	552 (1217) vs. 1925 (5786)	0.16	2239 (7923) vs. 1329 (4566)	0.47
TNM 1 vs. 3	384(1208) vs. 75 (198)	0.08	1856 (3095) vs. 1521(3114)	0.8	552 (1217) vs. 93 (217)	0.01	2239 (7923) vs. 137 (353)	0.04
TNM 1 vs. 4	384 (1208) vs. 1.5 (3)	0.02	1856 (3095) vs. 0.26 (0.53)	0.00	552 (1217) vs. 156 (33.7)	0.001	2239 (7923) vs. 0.004 (0.008)	0.03
TNM 2 vs. 3	180 (532) vs. 75 (198)	0.37	2328 (6035) vs. 1521 (3114)	0.61	1925 (5786) vs. 93 (217)	0.06	1329 (4566) vs. 137 (353)	0.13
TNM 2 vs. 4	180 (532) vs. 1.5 (3)	0.049	2328 (6035) vs. 0.26( 0.53)	0.03	1925 (5786) vs. 22.8 (33.7)	0.05	1329 (4566) vs. 0.004 (0.008)	0.09
TNM 3 vs. 4	75 (198) vs. 1.5 (3)	0.36	1521 (3114) vs. 0.26 (0.53)	0.24	93 (217) vs. 22.8 (33.7)	0.43	137 (353) vs. 0.004 (0.008)	0.35

Survival								
DF vs. LR	146 (566) vs. 959 (2223)	0.37	1551 (2851) vs. 1807 (2315)	0.79	994 (3987) vs. 776 (1311)	0.75	2110 (7490) vs. 271 (716)	0.04
DF vs. DR	146 (566) vs. 2.1 (3.3)	0.024	1551 (2851) vs. 10190 (14338)	0.25	994 (3987) vs. 372 (582)	0.23	2110 (7490) vs. 246 (500)	0.03
DF vs. D	146 (566) vs. 685 (1577)	0.23	1551 (2851) vs. 1190 (2787)	0.66	994 (3987) vs. 825 (1967)	0.81	2110 (7490) vs. 467 (1232)	0.07

SOCS 4 - 7 Mean mRNA expression levels in a cohort of 128 breast cancer patients; a comparison between subgroups with different tumour grade, NPI score, and TNM stage. SD: Standard deviation, DF: Disease free survival, LR: Local disease recurrence, DR: Distant disease recurrence, D: Death from breast cancer

After a median follow up of 10 years, SOCS4 mRNA expression levels were higher among women who remained disease free compared to those who developed distant recurrence [mean copy number 146 vs. 2.07, 95% CI (19, 269.6), p = 0.024].

After a median follow up of 10 years, there was a trend for tumours with higher SOCS4 expression levels to be associated with better OS (p = 0.007), and with marginal benefit in DFS although the later remained below statistical significance (p = 0.05). (Figures 1 and 2)

### SOCS5 (Table 4)

SOCS5 levels of expression were significantly lower in tumour tissue from patients with TNM-4 stage disease compared to normal background tissue; TNM-4 vs. background tissue [mean copy number 0.263 vs. 4318, 95% CI (-7867.49, -769), p = 0.019]. Similarly, SOCS5 mRNA expression was found to significantly decrease with increasing TNM stage; TNM-1 vs. TNM-4 [mean copy number 1856 vs. 0.263, 95% CI (1063, 2648.36), p = 0.0000] and TNM-2 vs. TNM-4 [mean copy number 2328 vs. 0.263, 95% CI (315, 4340.62), p = 0.025]. No prediction of better DFS or OS was found to be significantly associated with lower SOCS5 levels after 10 years follow up period.

### SOCS6 (Table 4)

SOC6 mRNA expression was found to significantly decrease with increasing tumour TNM stage: TNM-1 vs. TNM-3 [mean copy number 552 vs. 93, 95% CI (106, 812), p = 0.012]; and TNM-1 vs. TNM-4 [mean copy number 552 vs. 22.8, 95% CI (216, 843), p = 0.0013]. DFS and OS survival curves showed no significance of SOCS6 expression levels in predicting better survival after 10 years follow up period.

### SOCS7 (Table 4)

SOCS7 was found to be expressed in both normal/benign breast tissue and breast cancer specimens. No significant difference was found between SOCS7 expression in normal background tissue and its expression in breast cancer tissue.

The expression of SOCS7 mRNA did not significantly differ with increasing Nottingham Prognostic Index (NPI) or between normal background breast tissue and tumour tissues of patients with different NPI levels, but significantly decreased with increasing tumour grade; grade 2 vs. grade 3 [mean copy number 1396 vs. 427, 95% CI (62, 1876), p = 0.037].

The expression of SOCS7 mRNA was found to significantly decrease with increasing TNM stage; TNM-1 vs. TNM-3 [mean copy number 2239 vs. 137, 95% CI (56, 4148), p = 0.044], and TNM-1 vs. TNM-4 [mean copy number 2239 vs. 0.00375, 95% CI (209, 4268.3), p = 0.031].

SOCS7 transcript levels did not significantly differ with different tumour grades or with ER status.

After a median follow up of 10 years, we found SOCS7 mRNA expression levels to be higher among women who remained disease free compared to those who developed local recurrence [mean copy number 2110 vs. 271, 95% CI (99, 3580), p = 0.039]; and compared to those who developed distant metastasis [mean copy number 2110 vs. 246, 95% CI (149, 3578), p = 0.033]. SOCS7 expression was also higher among those who remained disease free compared to all other patients who developed any type of recurrence (local or distant) or died from breast cancer during the same follow up period [mean copy number 2110 vs. 372, 95% CI (40, 3436), p = 0.045].

There was a trend for tumours with lower SOCS7 expression levels to be associated with shorter DFS and OS times. The DFS and OS curves for women with tumours which were classified as having 'high levels' of SOCS7 transcript were found to differ significantly from those of their 'low level' counterparts. (Figures 1 and 2) The survival curves show that higher levels of SOCS7 are significant in predicting higher DFS (p = 0.03) and better OS (p = 0.035).

### Multivariate Analysis using Cox regression model

Table 5 summarizes the p value for the prognostic significance of the respective factors in predicting the overall survival and disease free survival, using Multivariate analysis based on the Cox regression model.

**Table 5:** Prediction of overall survival and disease free survival, using multivariate analysis based on Cox regression model.

PREDECTIVE FACTORS	OVERALL SURVIVAL (*p*)	DISEASE FREE SURVIVAL (*p*)
NPI	0.675	0.435
Grade	0.96	0.92
TNM	0.667	0.252
Nodal	0.092	0.504
SOCS1	0.025	0.089
SOCS2	0.785	0.37
SOCS3	0.76	0.91
SOCS4	0.32	0.739
SOCS5	0.55	0.258
SOCS6	0.043	0.374
SOCS7	0.024	0.083

A Summary of the p values for the prognostic significance of the respective factors in predicting overall survival and disease free survival, using multivariate analysis based on Cox regression model.

### SOCS3 and SOCS7 protein expression by immunohistochemistry

**Figure 3:**
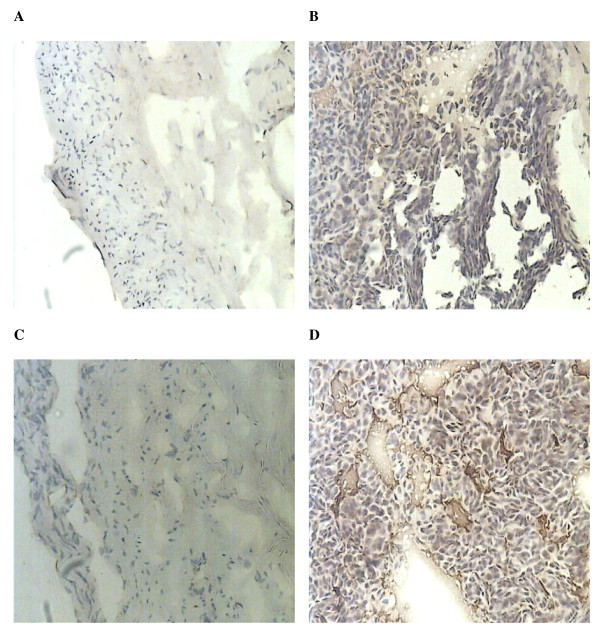
**Detection of SOCS3 and SOCS7 in primary breast cancer samples by immunohistochemistry**. Representative immunohistochemistry staining of samples with low SOCS3 (A), high SOCS3 (B), low SOCS7 (C), and high SOCS7 (D). (×100)

In 8 samples of primary breast cancer, immunohistochemical staining of SOCS3 and 7 correlated with their respective mRNA expression. In tumours with high SOCS3 and 7 mRNA expression levels, epithelial tumour cells showed cytoplasmic staining for the respective SOCS proteins (Figure 3). In normal mammary tissue, ducts, and stroma; cells exhibited staining for SOCS3 and 7 (not shown).

## Discussion

Several studies have investigated the role of SOCS protein family in the oncogenesis of several types of solid and haematological tumours indicating an important role of SOCS protein family in the tumour cellular growth and differentiation [29,37-42]. One important mechanism is SOCS gene promoter methylation which may result in SOCS gene silencing and subsequent loss of negative feedback control on the JAK/STAT signaling pathway in tumour cells [29,38-42]. Conversely, demethylation of SOCS genes promoters may result in restoration of SOCS mRNA and proteins expression, which in turn results in induction of apoptosis and suppression of growth [29].

Cytokine stimulation activates the JAK-STAT pathway, leading to induction of SOCSs. These SOCS proteins then inhibit the signaling pathways that STATs initially led to their production. SOCS proteins therefore act as part of a negative feedback loop. In various cancers, inhibition of STAT signaling - most significantly by SOCS proteins - suppresses cancer cell growth and induces apoptosis [29,43]. More data was particularly evident in SOCS1 and SOCS3 roles as tumor suppressors. In gastric cancer, for example, loss of SOCS 1 may be involved in lymph node metastasis and tumor progression [37], while restoration of its expression suppressed development and progression of hepatocellular carcinoma cells [44]. Similarly, SOCS3 may also be involved in the suppression of tumour growth and metastasis of several malignancies including lung cancer, hepatocellular cancer, and head and neck squamous cell carcinoma [45-47]. More recent studies showed that over-expression of SOCS3 markedly suppressed STAT3 expression, and inhibited STAT5 phosphorylation, resulting in decreased cell proliferation in T47D breast cancer cell lines, and decreased proliferation and anchorage-independent growth in MCF7 breast cancer cell lines[21]. Furthermore, the relative decreasing levels of SOCS expression in human breast tumours have been previously quantified and It was suggested an important local host defense mechanism during the progression period from *in situ *to invasive ductal carcinoma characterized by intense cytokine production from the lymphocytic infiltration. Such increased cytokine production as a part of this local mechanism induces SOCS protein over-expression [43]. Thus, a study by Camp et al performed on 89 human breast carcinomas showed strong lymphocytic production of Il-2, Il-4, TGF-β1, TNF-α as well as lower levels of IFNγ and GM-CSF [44]. These cytokines are major activators of SOCS/CIS signalling, particularly SOCS1 and 3 in granulocytes and lymphocytes [18,45].

In addition to the conventional inflammatory cytokines, PRL and GH - the major mammotrophic hormones - as well as IGF-I, may be involved in SOCS gene induction during breast carcinogenesis, and particularly known to induce substantial SOCS1-3 and CIS gene expression [23,46].

Therefore - and given the ubiquitous nature of SOCS gene expression in response to host cytokines - it is not unexpected that breast tumour tissue shows elevated SOCS expression [30].

Such over-expression of SOCS genes is thought to be a specific lesion in breast cancer tissue cells which may provide resistance to pro-inflammatory and trophic cytokines through blocking STAT signaling which mediates essential functions in the mammary tissue, and may well be implicated in mammary oncogenesis [47].

The above argument suggests a strong tumour suppressing role of SOCS proteins. However, little information exists regarding the significance of the association of various SOCS proteins expression with prognosis and clinico-pathological features of breast cancer.

In this study, we intended to elucidate the relationship between various clinical, pathological, prognostic and survival parameters and the expression of the SOCS family proteins 1 - 7 in patients with breast cancer using RT-PCR.

Our results show decreased SOCS 1,4,5,6 and 7 expression with increased TNM stage and decreased SOCS7 expression with higher tumour grade. These findings together with results from previous studies on SOCS 2 expression in breast cancer [30,31], and the observations from another report confirming higher expression levels of SOCS3 in tissues from patients with lymph node negative breast cancer [32]; strongly suggest a highly significant negative correlation between SOCS proteins and advancing clinico-pathological stage and poorer differentiation of breast carcinoma.

Furthermore, our results show decreased SOCS2 and 3 expressions with increased Nottingham Prognostic Index (NPI) which adds significant positive prognostic role of these proteins in breast cancer.

SOCSs mRNA expression (with the exception of SOCS5) showed no significant difference between cancer tissue samples and matched normal background tissue. Using histologically normal appearing samples as the sole control tissue is probably less appropriate. The use of donor tissues (ideally obtained under similar conditions as the tumor tissue) will serve as better controls. Donor control, in addition to normal adjacent to tumors, precancerous lesions, and tumor samples will provide the best sample set for resolution of genetic alterations that are relevant to the disease process by minimizing the potential implications of field cancerization. As there was no normal donor tissue used in this study, it was not clear whether the absence of a difference in SOCS expression between tumour and matched normal background tissue in our cohort was due to the effect of field cancerization.

Our data indicate that there may be a common signaling mechanism controlling the various SOCS proteins expression in breast cancer. Evidence from previous studies point to activated STAT5 as a possible SOCS2, SOCS3 and CIS genes controller and its loss may result in less differentiated and more malignant tumours, and subsequently; poorer prognosis [21,48-51]. Similar mechanism may well be existent for SOCS1 and SOCS4-7 gene control in breast cancer and this will need further investigation.

Our observations on the favorable role of SOCS protein family in several clinico-pathological and prognostic aspects of breast cancer have prompted us to perform survival analysis to explore possible correlation of SOCS1-7 proteins expression with disease-free survival and overall survival in our cohort of breast cancer patients.

After a median follow up period of 10 years, we found higher levels of SOCS1, 2 and 7 expression among those patients who remained disease-free compared to those who developed local recurrence.

Similarly, we found higher levels of SOCS2, 4, and 7 expression in those who remained disease-free compared to those who developed distant recurrence. Patients who remained disease-free had higher levels of SOCS1 and 2 expression compared to those who died from breast cancer.

The disease free survival and overall survival curves showed that higher levels of SOCS1, 3 and 7 were significant predictors of better disease free survival and overall survival. Higher levels of SOCS4 were significant in predicting better overall but not disease free survival.

Thus, in addition to the favorable positive correlation between higher mRNA expression of several members of the SOCS protein family and earlier more differentiated breast cancer, we have also demonstrated a significant positive correlation with patient's disease free and overall survival over a relatively long median follow up period.

## Conclusion

To our knowledge, this study is the first report to analyse the expression of 7 members of the SOCS family and to point to a favorable role of SOCS1, 3, 4, and 7 as predictors of earlier tumour stage and better prognosis and clinical outcome in breast cancer. Nevertheless, our understanding of the molecular mechanisms involved in the control of SOCS proteins expression and their regulation by various cytokines in normal and malignant breast tissue remains limited. Our data could be used in further validation studies in order to establish a clear mechanism of the JAK/STAT/SOCS interaction and its role in the development and progress of breast cancer. Further research should include correlations between SOCS genes expression and the expression of other genes related to apoptosis and proliferation.

## Competing interests

The authors declare that they have no competing interests.

## Authors' contributions

WS drafted the manuscript and carried out part of the molecular genetic work; WGJ supervised the PCR and IHC experiments and the quantitative analysis; AS contributed to the design of the study and writing of the manuscript and surgically excised the cohort tissue samples; and KM conceived the study, participated in its design and coordination, and surgically excised the cohort tissue samples.

All authors read and approved the final manuscript.

## Pre-publication history

The pre-publication history for this paper can be accessed here:

http://www.biomedcentral.com/1471-2407/10/178/prepub
